# Safety of Custodiol for Myocardial Protection in Minimally Invasive Mitral Valve Repair: A Japanese Single-Center Retrospective Comparison with Blood Cardioplegia in Conventional Sternotomy Repair

**DOI:** 10.5761/atcs.oa.25-00208

**Published:** 2026-02-21

**Authors:** Hiroaki Aizawa, Akihiro Yoshitake, Yuki Katsunori, Osamu Kinoshita, Yu Kumagai, Taro Kuroda, Yuta Kanazawa, Yuko Gatate, Takayuki Gyoten, Toshihisa Asakura

**Affiliations:** 1 Department of Cardiovascular Surgery, Saitama Medical University International Medical Center, Hidaka, Saitama, Japan; 2 Department of Clinical Engineering, Saitama Medical University International Medical Center, Hidaka, Saitama, Japan

**Keywords:** minimally invasive mitral valve repair, Custodiol cardioplegia, Bretschneider HTK solution, myocardial protection

## Abstract

**Purpose:**

Research regarding Custodiol’s safety in minimally invasive mitral valve repair remains limited in Asian populations. We compared Custodiol in minimally invasive mitral valve repair to repetitive cold blood cardioplegia in open mitral valve repair.

**Methods:**

We retrospectively evaluated 98 consecutive patients who underwent minimally invasive mitral valve repair with Custodiol and 70 consecutive patients who underwent open mitral valve repair with repetitive cold blood cardioplegia at our institution between January 2015 and December 2024. The primary endpoints were creatine kinase-myocardial band (MB) levels and left ventricular ejection fraction determined by echocardiography pre- and post-surgery.

**Results:**

Maximum creatine kinase-MB levels within 48 h post-surgery were significantly lower in the minimally invasive group than in the open repair group, both in the overall cohort (45.0 vs. 60.7 U/L; *p* <0.001, respectively) and after excluding patients who underwent Maze procedure or pulmonary vein isolation (42.4 vs. 50.0 U/L; *p* = 0.009, respectively). Left ventricular ejection fraction pre- and post-surgery was comparable between the minimally invasive and open repair groups (72% vs. 69%; *p* = 0.426 and 59% vs. 60%; *p* = 0.204, respectively).

**Conclusion:**

Custodiol during minimally invasive mitral valve repair provides myocardial protection comparable to repetitive cold blood cardioplegia in open mitral valve repair.

## Introduction

Myocardial protection is crucial in cardiac surgery and has evolved alongside advancements in modern cardiac surgery. Cardioplegia, employed for myocardial protection, can be categorized into 2 primary types based on extracellular components (e.g., blood cardioplegia) or intracellular electrolytes.^1)^ Custodiol (Bretschneider HTK solution; Koehler Chemie, Bensheim, Germany) is an intracellular crystalloid cardioplegic solution containing a low sodium concentration, which prevents myocyte depolarization and sodium channel inactivation.^2)^ Custodiol is particularly advantageous in minimally invasive cardiac surgery (MICS) owing to its requirement for only single antegrade administration, along with the provision of myocardial protection for over 160 min of cross-clamp time, in contrast to the intermittent delivery required by repetitive cold blood cardioplegia.^3)^ Single-dose delivery of cardioplegia does not disturb the technical flow or surgical field. Comparative studies of Custodiol and intermittent myocardial protection have been conducted since the 1980s, with numerous reports published since the 2000s, particularly in the field of open-heart surgery, confirming the safety of Custodiol solution.^4,5)^ However, there is a paucity of studies evaluating the safety of Custodiol solution in MICS, specifically in mitral valve repair surgery, comparing the safety of Custodiol solution with that of repetitive cold blood cardioplegia in open-heart mitral valve repair.^6)^ Few studies have examined the safety of myocardial protection using Custodiol in MICS compared to that of open-heart mitral valve surgery, particularly concerning perioperative cardiac function and cardiac biomarkers; however, such evidence remains scarce in Asian cohorts, in which Custodiol is not yet widely used. In the present single-center retrospective study, we aimed to assess the safety and feasibility of a single dose of Custodiol in minimally invasive mitral valve repair (MIMVr) compared to repetitive cold blood cardioplegia in open mitral valve repair (OMVr).

## Materials and Methods

### Population and study design

This retrospective observational study adhered to the Declaration of Helsinki and was approved by the ethics committee of the Saitama Medical University International Medical Center (approval number: 2025-009). Given the retrospective nature of this study, patients were provided with opt-out participant information instead of written informed consent. Data were manually extracted from patient records. This study screened 104 consecutive patients who underwent MIMVr using Custodiol (Bretschneider HTK solution; Koehler Chemie, Bensheim, Germany) between January 2015 and December 2024 at the Saitama Medical University International Medical Center. Of these, 3 patients who underwent combined mitral and tricuspid procedures were excluded. In addition, 3 patients who required conversion to median sternotomy were also excluded. Consequently, 98 patients were included in the MIMVr group. Additionally, 315 consecutive patients who underwent OMVr using repetitive cold blood cardioplegia at our institution during the same period were screened. Of these, 245 patients who underwent repeat surgery, morrow operation, aortic surgery, left ventricular assist device surgery, combined coronary artery bypass surgery, combined mitral and tricuspid procedures, or combined mitral and aortic valve surgery were excluded. Patients undergoing concurrent Maze procedure, pulmonary vein isolation (PVI), left atrial appendage closure or resection, and patent foramen ovale closure were not excluded from either group. As a result, 70 patients were included in the OMVr group.

### Operative details and cardioplegia administration

The operative details relevant to myocardial protection in MIMVr at our institution were as follows: Peripheral cardiopulmonary bypass was established via right femoral arterial, femoral venous, and internal jugular venous cannulations, with systemic temperature reduced to 32°C–34°C. A dual-lumen aortic root cannula (ANTE FLEX; Senko Medical Instrument Mfg. Co., Ltd., Tokyo, Japan) was inserted into the ascending aorta. Following aortic cross-clamping, 1600 mL of Custodiol antegrade cardioplegia at 4°C–6°C was administered, with the infusion pressure adjusted based on the aortic root pressure, with target pressures of 80–100 mmHg before cardiac arrest and 50–70 mmHg after cardiac arrest. No cases of moderate or severe aortic insufficiency were observed in the MIMVr group. Custodiol (1000 mL) was administered if electrical or mechanical activity was observed. When aortic cross-clamping was anticipated to exceed 150 min, an additional 1000 mL of Custodiol was administered. In cases requiring a second pump run post-aortic declamping, 1600 mL of Custodiol antegrade cardioplegia was utilized for repetitive administration. As for the OMVr group, all patients underwent a median sternotomy for OMVr. Cardiopulmonary bypass was established via bicaval cannulation of the superior vena cava, inferior vena cava, and ascending aorta cannulations, with the systemic temperature reduced to 34°C. Initially, 1500 mL of blood induction cardioplegia, comprising myocardial protection (MIOTECTER, Fuso Pharmaceutical Industries, Ltd., Osaka, Japan) and blood (mixed at a 2:1 ratio of cardioplegic solution to blood), with potassium chloride added at a dose of 5 mEq per 500 mL of MIOTECTER, was administered through the antegrade route. Following the initial induction, blood maintenance cardioplegia mixed at a 1:1 ratio (cardioplegic solution:blood) was infused for 4 min every 20–25 min at a rate of 200–300 mL/min, maintaining adequate perfusion pressure (20–40 mmHg) in a retrograde fashion. For cases in which the antegrade method was employed for repeated cardioplegia administration, 1000 mL of blood maintenance cardioplegia mixed at a 1:1 ratio (cardioplegic solution:blood) was infused every 20–25 min. A hotshot retrograde dose was administered prior to myocardial reperfusion. The calculated components of the cardioplegia solutions used in the procedures are detailed in **Table 1**.

**Table 1:** Calculated composition of the cardioplegia solutions used in the present study

Component	Custodiol	Blood induction cardioplegia	Blood maintenance cardioplegia	Units
Na^+^	15	126.0–128.3	129.0–132.5	mmol/L
K^+^	9	18.5–18.9	9.8–10.4	mmol/L
Mg^2+^	4	10.9–11.0	8.4–8.5	mmol/L
Ca^2+^	0.015	1.5–1.6	1.7–1.9	mmol/L
Histidine	198	–	–	mmol/L
Tryptophan	2	–	–	mmol/L
Ketoglutarate	1	–	–	mmol/L
Mannitol	30	–	–	mmol/L
Glucose	–	1.4–2.0	2.0–3.0	mmol/L
Lidocaine	–	–	–	mg/L
pH	7.02–7.20	7.10–7.20	7.18–7.25	–

### Definitions

The preoperative mitral regurgitation (MR) grade was determined based on transthoracic echocardiography results obtained within 30 days pre-surgery. The degree of postoperative MR was assessed by transthoracic echocardiography on the seventh postoperative day. Postoperative complications were defined as any deviations from the normal postoperative course. Myocardial infarction was defined as the presence of at least 2 of the following: cardiac enzyme elevation (creatine kinase-myocardial band [CK-MB] ≥100 U/L), new regional ventricular wall motion abnormality on echocardiography, and new pathologic waves on an electrocardiogram. In-hospital mortality included any death occurring within 30 days post-surgery or during the same hospital admission period as the operation. Postoperative mechanical circulatory support was defined as the use of an intra-aortic balloon pump, extracorporeal membrane oxygenation, or Impella device. The year of surgery was divided into 2 periods: 2015–2019 (January 2015–December 2019) and 2020–2024 (January 2020–December 2024).

### End points

The primary endpoints were CK-MB levels (value at intensive care unit [ICU] admission and maximum level within 48 h post-surgery) and left ventricular ejection fraction (LVEF) based on transthoracic echocardiography pre- and post-surgery. As the Maze procedure and PVI could cause myocardial damage, CK-MB levels were also analyzed after excluding patients who underwent the Maze procedure or PVI. The secondary endpoints were the rate of postoperative complications within 30 days post-surgery, in-hospital mortality, length of ICU stay, and length of hospital stay.

### Statistical analysis

Statistical analyses were conducted using JMP Pro (version 17.0; SAS Institute Inc., Cary, NC, USA). Normality of all continuous variables was assessed using the Shapiro–Wilk test. Normally distributed variables are reported as mean ± standard deviation, whereas non-normally distributed variables are summarized as median with the 25th–75th percentile interquartile range (IQR). Categorical variables are expressed as counts and percentages. Unpaired continuous variables with a normal distribution were compared using the Student’s *t*-test, and unpaired continuous variables with a non-normal distribution were compared using the Mann–Whitney U test. Categorical data were compared using the chi-squared test or Fisher’s exact test. Odds ratios with corresponding 95% confidence intervals were estimated using logistic regression to identify predictors of maximum postoperative CK-MB level (≥100 U/L) within 48 h after surgery. Variables considered clinically relevant and showing a *p*-value <0.20 in univariable analyses were regarded as candidate covariates and entered into a multivariable logistic regression model using a backward stepwise selection approach. For continuous variables, odds ratios are expressed per 1-unit increase based on the original scale of each variable. Statistical significance was defined as *p* <0.05 for all analyses. In the OMVr group, 1 value was missing from the postoperative LVEF based on transthoracic echocardiography. The incidence of missing data in our models was minimal, and no imputation was performed for missing data.

## Results

### Patient characteristics and operative data

A total of 98 patients underwent MIMVr using a single dose of Custodiol (MIMVr group), and 70 patients underwent OMVr using repetitive cold blood cardioplegia (OMVr group). The patient characteristics of the 2 groups are summarized in **Table 2**, and operative data are presented in **Table 3**. Fourteen and 23 patients underwent concomitant Maze procedure or PVI in the MIMVr and OMVr groups, respectively. In the MIMVr group, 14 patients required repeated doses of cardioplegic agents. The specific reasons for repeat cardioplegia in the MIMVr group are as follows: 7 patients required a second aortic cross-clamp for the purpose of repeat intervention on the mitral valve, 1 patient required a second aortic cross-clamp because of bleeding from the pacing site, 5 patients had electrical activity within 15 min of the first dose of cardioplegia, and 1 patient required prolonged aortic cross-clamping time (anticipated to exceed 150 min). The longest single aortic cross-clamp without repeat cardioplegia was 158 min. No patients required a third dose of Custodiol.

**Table 2:** Demographic and preoperative clinical characteristics of patients in the MIMVr and OMVr groups

Variables	MIMVr (n = 98)	OMVr (n = 70)	*p*-Value
Male	66 (67%)	44 (63%)	0.547
Age (years)	59 (52–67)	64 (57–73)	0.006
BSA (m^2^)	1.63 ± 0.19	1.60 ± 0.18	0.320
BMI (kg/m^2^)	22.5 (20.1–24.4)	22.6 (18.9–25.3)	0.915
EuroSCORE II (%)	0.68 (0.56–0.84)	0.77 (0.63–0.98)	0.002
NYHA III, IV	2 (2%)	6 (9%)	0.068
Hemoglobin level (g/dL)	13.5 ± 1.5	13.0 ± 2.1	0.070
Preoperative creatinine level (mg/dL)	0.78 (0.67–0.90)	0.85 (0.68–1.09)	0.116
Hemodialysis	0 (0%)	2 (3%)	0.172
Infective endocarditis	4 (4%)	11 (16%)	0.013
Diabetes	4 (4%)	15 (21%)	0.001
Hypertension	22 (22%)	29 (41%)	0.008
Previous myocardial infarction	1 (1%)	5 (7%)	0.083
Hyperlipidemia	12 (12%)	14 (20%)	0.171
Cerebrovascular disease	8 (8%)	10 (14%)	0.206
Chronic obstructive pulmonary disease	0 (0%)	2 (3%)	0.172
Atrial fibrillation	13 (13%)	24 (34%)	0.001
Severity of mitral valve regurgitation			
Moderate-to-severe	5 (5%)	9 (13%)	0.073
Severe	93 (95%)	61 (87%)	0.073
Mitral valve prolapse involved			
Anterior leaflet	16 (16%)	24 (34%)	0.007
Posterior leaflet	77 (79%)	35 (50%)	<0.001
Bi-leaflets	5 (5%)	11 (16%)	0.021
Year of surgery			
2020–2024	56 (57%)	22 (31%)	0.001

MIMVr: minimally invasive mitral valve repair; OMVr: open mitral valve repair; BSA: body surface area; BMI: body mass index; NYHA: New York Heart Association

**Table 3:** Operative characteristics of patients undergoing MIMVr and OMVr

Variables	MIMVr (n = 98)	OMVr (n = 70)	*p*-Value
Elective	98 (100%)	69 (99%)	0.417
Mitral valve repair only	82 (84%)	42 (60%)	0.001
Annuloplasty	95 (97%)	65 (93%)	0.280
Concomitant procedure			
Maze procedure or pulmonary vein isolation	14 (14%)	23 (33%)	0.004
Left atrial appendage closure or resection	13 (13%)	15 (21%)	0.162
Patent foramen ovale closure	1 (1%)	2 (3%)	0.571
Administration of a repeat dose of cardioplegia	14 (14%)	70 (100%)	<0.001
Second aortic cross clamp	8 (8%)	2 (3%)	0.197
Procedure time (min)	275 ± 50	266 ± 60	0.311
Cardiopulmonary bypass time (min)	146 (131–167)	131 (111–162)	0.022
Aortic cross clamp time (min)	114 (94–130)	104 (87–123)	0.130
No intraoperative blood transfusion	77 (79%)	39 (56%)	0.002
Lowest core temperature under extracorporeal circulation (°C)	34.0 (33.6–34.2)	34.1 (34.0–34.3)	0.001

MIMVr: minimally invasive mitral valve repair; OMVr: open mitral valve repair

### Primary endpoints

The results of the primary endpoints are listed in **Table 4**. CK-MB levels were significantly lower in the MIMVr group than in the OMVr group at ICU admission (*p* <0.001) and at their maximum within 48 h post-surgery (*p* <0.001). These differences remained significant even after excluding patients who underwent the Maze or PVI procedures. Regarding left ventricular function, preoperative LVEF did not differ between the 2 groups (*p* = 0.426). Postoperative LVEF was also comparable between the MIMVr and OMVr groups (*p* = 0.204).

**Table 4:** Primary endpoints of the study with LVEF and CK-MB values in the MIMVr and OMVr groups

Variables	MIMVr	OMVr	*p*-Value
Preoperative LVEF (%)	72 (65–77)	69 (61–78)	0.426
Postoperative LVEF (%)	59 (51–67)	60 (54–71)	0.204
CK-MB levels on ICU admission (U/L)	42.8 (29.3–64.1)	62.1 (41.2–112.1)	<0.001
CK-MBmax during 48 h after surgery (U/L)	45.0 (30.6–64.1)	60.7 (41.2–112.1)	<0.001
**Variables excluding Maze or pulmonary vein isolation cases**	**MIMVr (n = 84)**	**OMVr (n = 47)**	***p*-Value**
CK-MB levels on ICU admission (U/L)	39.2 (27.0–51.6)	50.0 (33.4–68.8)	0.001
CK-MBmax during 48 h after surgery (U/L)	42.4 (29.0–54.1)	50.0 (33.4–68.8)	0.009

LVEF: left ventricular ejection fraction; CK-MB: creatine kinase-myocardial band; MIMVr: minimally invasive mitral valve repair; OMVr: open mitral valve repair; ICU: intensive care unit

### Secondary endpoints and postoperative outcomes

The postoperative outcomes, including the secondary endpoints, are summarized in **Table 5**. The incidence of postoperative complications within 30 days post-surgery were 7% (n = 7) and 9% (n = 6) in the MIMVr and OMVr groups, respectively (*p* = 0.733). In the MIMVr group, complications included postoperative chest wall bleeding requiring re-exploration (n = 3), aspiration pneumonia (n = 1), re-expansion pulmonary edema (n = 1), grade 1 right-sided pneumothorax (n = 1), and stroke (n = 1); all patients were subsequently discharged. In the OMVr group, complications included non-occlusive mesenteric ischemia (n = 1), spontaneous perforation of the colon (n = 1), sick sinus syndrome (n = 1; discharged without pacemaker implantation), second-degree atrioventricular block (Wenckebach) and pancreatitis (n = 1), femoral arterial thrombosis requiring Fogarty balloon catheter embolectomy caused by migrated vegetation after the surgery for infective endocarditis (n = 1), and radial nerve injury (n = 1). The patient who developed non-occlusive mesenteric ischemia as a postoperative complication died 8 days post-surgery because of multiple organ failure. The patient with spontaneous perforation of the colon underwent colon resection and was transferred to another hospital for rehabilitation in stable condition 57 days post-OMVr. The remaining 4 patients were discharged in stable condition. In-hospital mortality did not differ significantly between groups (MIMVr group: 0 (0%) patients; OMVr group: 1 (1%) patient; *p* = 0.417). The length of ICU stay was comparable between the groups (MIMVr group: 3 (IQR: 2–4) days, OMVr group: 3 (IQR: 2–5) days; *p* = 0.009). Aortic cross-clamp times did not differ significantly between groups (*p* = 0.130). The rate of surgeries performed without blood transfusion was higher in the MIMVr group (79%; n = 77) than in the OMVr group (56%; n = 39; *p* = 0.002). Successful mitral valve repair with no, trivial, or mild residual MR on postoperative echocardiographic assessment was observed in 98% and 99% of the MIMVr and OMVr groups, respectively (*p* = 1.000).

**Table 5:** Postoperative outcomes for patients undergoing MIMVr and OMVr

Variables	MIMVr (n = 98)	OMVr (n = 70)	*p*-Value
Postoperative complications within 30 days of surgery (%)	7 (7%)	6 (9%)	0.733
In-hospital mortality (%)	0 (0%)	1 (1%)	0.417
Length of ICU stay (days)	3 (2–4)	3 (2–5)	0.009
Hospital stay (days)	11 (10–14)	15 (11–19)	<0.001
Re-exploration for bleeding	3 (3%)	0 (0%)	0.267
Postoperative myocardial infarction	0 (0%)	0 (0%)	1.000
Pacemaker implantation	0 (0%)	0 (0%)	1.000
Use of mechanical circulatory support	0 (0%)	0 (0%)	1.000
Postoperative max creatinine level (mg/dL)	0.84 (0.75–1.00)	0.99 (0.77–1.20)	0.015
Postoperative mitral regurgitation grade			
None–mild	96 (98%)	69 (99%)	1.000
Moderate	2 (2%)	1 (1%)	1.000

MIMVr: minimally invasive mitral valve repair; OMVr: open mitral valve repair; ICU: intensive care unit

### Predictors of postoperative CK-MB elevation

Univariable and multivariable logistic regression analyses were performed to assess the influence of preoperative characteristics on a maximum postoperative CK-MB level ≥100 U/L (**Table 6**). In the overall cohort, year of surgery (2020–2024) and concomitant Maze or PVI procedures remained independently associated with CK-MB elevation. Among patients who underwent MIMVr with concomitant Maze or pulmonary vein isolation procedures, 13 of 14 cases were performed during the 2020–2024 period. Based on this distribution, subsequent analyses were performed after excluding patients who underwent concomitant Maze or PVI procedures (**Table 4**).

**Table 6:** Predictors of maximum postoperative CK-MB level (≥100 U/L) within 48 h after surgery in the overall cohort

Variables	Univariable analysis		Multivariable analysis	
	OR (95% CI)	*p*-Value	OR (95% CI)	*p*-Value
Group				
MIMVr group	0.45 (0.21–0.96)	0.039		
OMVr group	Reference			
Age (years)	1.02 (0.99–1.05)	0.262		
EuroSCORE II (%)	0.73 (0.19–1.13)	0.486		
Infective endocarditis	0.25 (0.01–1.31)	0.188		
Diabetes	1.02 (0.27–3.03)	0.980		
Hypertension	1.26 (0.56–2.74)	0.570		
Atrial fibrillation	16.29 (6.86–41.17)	<0.001		
Mitral valve prolapse involved				
Anterior leaflet	1.64 (0.70–3.69)	0.237		
Posterior leaflet	0.59 (0.28–1.29)	0.182		
Bi-leaflets	1.30 (0.35–4.03)	0.667		
Year of surgery				
2020–2024	2.72 (1.27–6.08)	0.012	3.24 (1.13–10.25)	0.028
2015–2019	Reference		Reference	
Maze procedure or pulmonary vein isolation	41.5 (15.77–122.66)	<0.001	44.53 (16.18–142.18)	<0.001

CK-MB: creatine kinase-myocardial band; MIMVr: minimally invasive mitral valve repair; OMVr: open mitral valve repair; OR: odd ratio; CI: confidence interval

## Discussion

We evaluated the perioperative cardiac biomarkers and LVEF using echocardiography to confirm the perioperative safety and feasibility of single-dose Custodiol cardioplegia in MIMVr. Our findings demonstrate that the MIMVr group utilizing Custodiol solution achieved satisfactory outcomes in absolute terms and was non-inferior to the OMVr group using repetitive cold blood cardioplegia. In Japan, MICS was first reported in the early 1990s, but there were few academic reports on MICS in Japan, even in the early 2000s. The landscape regarding MICS has evolved dramatically in recent years with the founding of the Japanese Association for Minimally Invasive Cardiac Surgery in 2015, and insurance reimbursement in 2018.^7)^ The early- and mid-term safety outcomes of MIMVr surgery in Japan have also improved in recent years.^8)^ As MICS has gained popularity, interest in myocardial protection strategies has increased. In particular, with regard to cardioplegia, a single- or dual-dose strategy using Custodiol or del Nido solutions for myocardial cardioplegia is appealing, having become indispensable for maintaining the operating fields in MICS surgery.^2)^ Custodiol is particularly suitable for MICS because it provides better myocardial protection than the del Nido solution during prolonged aortic cross-clamping procedures.^9)^ The low sodium content of Custodiol facilitates a high concentration of histidine, which provides a high buffering capacity for addressing ischemia-induced acidosis.^2,6)^ Ketoglutarate enhances ATP production during reperfusion, while tryptophan stabilizes the cell membranes, and mannitol reduces cellular edema by maintaining osmolality.^4)^ A systematic review by Edelman et al. revealed that in the case of myocardial protection in adult cardiac surgery, using Custodiol is comparable to standard myocardial cardioplegia (blood or crystalloid) regarding cardiac enzymes, low cardiac output syndrome, perioperative myocardial infarction, and postoperative mortality.^4)^

In terms of cardiac enzymes, CK-MB levels were significantly lower in the MIMVr group, even after excluding patients who underwent the Maze procedure or PVI. There were no significant differences in pre- and postoperative LVEF between the MIMVr and OMVr groups. These findings indicate that the Custodiol solution group was non-inferior to the OMVr group. From the perspective of cardioplegia using cardiac biomarkers and LVEF, similar conclusions were reported by Vivacqua et al.^2)^ They conducted a prospective randomized trial that compared 55 patients receiving MICS using repetitive cold blood cardioplegia and 55 patients receiving MICS using Custodiol, and reported no significant differences in terms of CK-MB, preoperative LVEF, and postoperative LVEF.

Unlike randomized trials, our retrospective cohort differed with respect to patient backgrounds. Given these imbalances, univariable and multivariable logistic regression analyses were performed using a maximum postoperative CK-MB level ≥100 U/L within 48 h after surgery as the outcome. In univariable analysis, group assignment (MIMVr group vs. OMVr group) was one of the factors associated with CK-MB level ≥100 U/L. In multivariable analysis, concomitant Maze or PVI procedures and operative era (2020–2024) remained independent predictors, whereas group assignment was no longer statistically significant. Notably, after excluding patients who underwent Maze or PVI, the year of surgery was no longer significantly associated with postoperative CK-MB level ≥100 U/L, suggesting that atrial fibrillation procedures partly influenced this finding.

Regarding postoperative clinical courses, the hospital stay was significantly shorter in the MIMVr group, whereas the ICU stay was comparable between the groups. These findings are consistent with a previous study, which reported that both ICU and hospital stays were significantly shorter in the MIMVr group using Custodiol than in the median sternotomy group using repetitive blood cardioplegia.^6)^ Our outcomes are acceptable in absolute terms as previous reports showed that the average ICU stay was 3.1 days and the average hospital stay was 10.6 days following mitral valve plasty or replacement^10)^ However, these favorable outcomes cannot be attributed solely to myocardial protection with Custodiol, as differences in surgical approach between MIMVr and OMVr may have influenced the length of ICU and hospital stay. Regarding blood transfusion, our study revealed that the number of surgical procedures performed without blood transfusion was significantly higher in the MIMVr group than in the OMVr group. Previous research revealed lower intraoperative transfusion rates in the MICS group with a single dose of myocardial protection than in the median sternotomy group with intermittent myocardial protection.^6)^ The total volume of cardioprotective solution administered tended to be higher in the repetitive myocardial protection group than in the Custodiol group, potentially inducing hemodilution due to high-volume cardioplegia. In addition, bleeding related to median sternotomy, particularly sternal bleeding, represents another major contributor to intraoperative transfusion requirements in the OMVr group.

While other variables demonstrated improvement, there were more re-exploration cases in the MIMVr group than in the OMVr group, although the difference was not statistically significant. This outcome did not adversely affect the postoperative complication rate, length of hospital stay, or in-hospital mortality. In this study, only 1 case received an additional Custodiol due to the anticipation of prolonged aortic cross-clamping. Therefore, we believe that further studies are needed to investigate optimal strategies and safety for additional Custodiol administration in cases of prolonged aortic cross-clamping.

This study has limitations that must be acknowledged. First, the retrospective nature of the analysis may have introduced selection bias. Second, due to differences in patient background factors (age, EuroSCORE II, infective endocarditis, diabetes, hypertension, atrial fibrillation, and location of mitral valve prolapse) between the MIMVr and OMVr groups, the 2 groups were not entirely comparable. Third, our comparison simultaneously differed by surgical approach (MIMVr vs. OMVr) and cardioplegia strategy (Custodiol vs. repetitive cold blood cardioplegia). Therefore, the independent effect of Custodiol cannot be fully isolated, and confounding by surgical approach must be considered. In particular, perioperative outcomes such as intraoperative blood transfusion requirements and postoperative recovery may be strongly influenced by differences related to the surgical approach itself. Consequently, prospective trials with larger cohorts are required to mitigate these biases. Ideally, such studies should compare Custodiol with blood cardioplegia within the same surgical approach, specifically MIMVr, to isolate the effect of the solution itself. To our knowledge, a retrospective study, particularly in Asian populations, examining the safety of Custodiol in MIMVr compared to OMVr using repetitive cold blood cardioplegia from the perspective of perioperative cardiac biomarkers and LVEF based on echocardiography has not been previously reported. We assert that our analysis provides important insights into the safety of Custodiol in MICS, thereby serving as a foundation for future prospective trials directly comparing Custodiol and blood cardioplegia within the same MIMVr approach.

## Conclusion

The administration of Custodiol during MIMVr offers satisfactory myocardial protection, comparable to the use of repetitive cold blood cardioplegia during OMVr in terms of cardiac enzymes and LVEF.

## Declarations

### Data availability

The data supporting the findings of this study are available from the corresponding author upon reasonable request. The data are not publicly available due to privacy and ethical restrictions.
